# Spray-Drying to Get Spin-Crossover Materials

**DOI:** 10.3390/ma10010060

**Published:** 2017-01-11

**Authors:** Nathalie Daro, Lucie Moulet, Nicolas Penin, Nicolas Paradis, Jean-François Létard, Eric Lebraud, Sonia Buffière, Guillaume Chastanet, Philippe Guionneau

**Affiliations:** CNRS, University of Bordeaux, ICMCB, UPR9048, 87 Avenue du Docteur Schweitzer, F-33600 Pessac, France; nathalie.daro@icmcb.cnrs.fr (N.D.); mouletlucie@gmail.com (L.M.); nicolas.penin@icmcb.cnrs.fr (N.P.); nicolas.paradis33@gmail.com (N.P.); letard@olikrom.com (J.-F.L.); eric.lebraud@icmcb.cnrs.fr (E.L.); sonia.buffiere@icmcb.cnrs.fr (S.B.); guillaume.chastanet@icmcb.cnrs.fr (G.C.)

**Keywords:** spin crossover, spray-drying, nanoparticles, iron, triazole, molecular switch, crystallite

## Abstract

Spin-crossover (SCO) triazole-based coordination polymers can be synthesized by micelle techniques, which almost always lead to rod-shaped nanoparticles. In order to notably reach new morphologies, we explore here the potentiality of the spray-drying (SD) method to get SCO materials. Three SCO coordination polymers and a mononuclear complex are investigated. In all cases, the SD method obtains particles definitely showing SCO. The features of the latter are yet always different from those of the referenced materials, in the sense that SCO is more gradual and incomplete, in adequacy with the poor crystallinity of the powders obtained by SD. In the case of coordination polymers, the particles are preferentially spherical. Indications of possible polymorphism and/or new materials induced by the use of the SD method are evidenced. In the case of the mononuclear complex, the SD method has allowed reproducing, in a quick and easy way, the well-known bulk compound. This exploratory work demonstrates the relevance of the concept and opens the way to a systematic scrutiny of all the experimental parameters to tune the size, morphology, and properties of the SD-synthesized SCO particles.

## 1. Introduction

The design of spin-crossover (SCO) nanoparticles (NPs) lies at the crossroads of fundamental interests and applicative targets. The thermal-induced SCO phenomenon on micro- and macro-samples is widely studied and well understood, even though some fundamental aspects are obviously still in discussions [1,2,3], but reducing the size of the sample to the nanoscale has opened numerous new questions and opportunities [4,5,6,7]. Among them are, for example, the effects of the size reduction on the SCO features (temperature, hysteresis width, converted fraction, etc.) as well as the relation between the SCO NPs’ morphologies and their physical properties. Many applicative targets of SCO materials are identified in fields as diverse as solid-state electronic devices (such as time-temperature integrators or high-density storage), X-chromic pigments, mechanical actuators, and optical sensors [4,5,6,7,8,9,10,11,12,13]. However, building SCO-based functional devices requires the use of NPs easily obtained and with controlled sizes and morphologies. Consequently, routes to get SCO NPs must be paved in a reliable, reproducible, and efficient way, which can be considered as a relatively recent topic since the first attempts come from the last decade [14,15]. In the race to the design of SCO NPs, it is usually aimed to reduce the volume of the sample to the nanoscale, keeping the same SCO features and particle morphology as in bulk materials. In the present work, we wish to completely reverse the target and to synthesize SCO particles using a new route, having in mind the design of innovative particles morphologies.

Despite not being exclusive, main efforts to grow SCO NPs have been performed on Fe (II) coordination polymers of the [Fe(R-trz)_3_]·X_2_ family (where R-trz = triazole stands for 4-R-substituted-1,2,4-triazole), since they undergo cooperative thermally induced SCO transitions near room temperature and are associated to a net change of color from pink to white [7,8,14,15,16,17,18,19]. Contrary to old statements, these compounds are generally well crystallized. Single-crystal and powder X-ray structural analyses show that the solid cohesion is based on 1D [Fe(R-trz)_3_] chains that interact directly or through the counter anions and present a strong reversible length reduction (about 10%) at the SCO [20,21,22,23,24]. The latter corresponds to a strong anisotropy of the SCO mechanical effects on the particles. In this family of SCO materials, the reverse micelle technique is successfully used to get NPs down to a few nanometers in size [14,15,25,26]. SCO features, including hysteresis width, are almost kept—although this point is debated for very small NPs—and in some cases the particles adopt a rod morphology. The size of the nanorods, especially their length, can even been controlled by playing with some parameters of the micelle synthesis protocol [27]. The functionalization of these kinds of SCO NPs is presently underway and, for example, hybrid particles represent a possible path to overcome the fragility of these NPs or to enhance their switching features. In this context, for example, functionalized [Fe(R-trz)_3_] particles of SCO@SiO_2_@Au [28,29] and SCO@Au [30] have been recently synthesized based on micelle techniques or by integration in mesoporous silica for SCO@MCM [31]. Since these approaches always result in nanorods showing the bulk-material SCO properties, a new approach has to be used to get to new morphologies of SCO particles. Other methods based on resin also lead to particles with nanorod morphologies [32]. In order to have a chance to reach innovative NPs morphologies and possibly novel properties within this family of SCO triazole-based coordination polymers, we explore here the use of the spray-drying method.

Spray-drying (SD) is a method to transform aqueous solution into powders that is commonly used by food and pharmaceutical industries since it is quick, cheap, and belongs to green chemistry. In academic research, spray-drying is used to synthesize materials and to control the morphologies of particles [33,34]. Our compounds are, by definition, thermosensitive, and the fact that particles are not exposed to high temperature is a priori one of the advantages of the spray-drying process in comparison to other existing drying processes. As a general matter, SD is known to preferentially lead to homogenous powders of spherical NPs [35]. Among the latter, many variations are encountered, from purely spherical to donutlike morphologies, including dense, hollow, hairy, or porous particles [34]. Interestingly, in many cases, the final morphology can be controlled by the experimental parameters [36,37]. In addition, SD can also be used to obtain nanocomposite particles, including encapsulated ones. For example, γ-Fe_2_O_3_@SiO_2_ and Fe_2_O_3_@graphen NPs were successfully designed by SD leading to enhancement of the magnetic properties [38,39]. Elsewhere, one of the main present challenges in the SCO field is to grow thin films, which could be achieving using SD methods [40]. This context seems to clearly indicate that SD could fulfill some of the expectations in the field of the simple and efficient design of new SCO NPs.

In the present study, the SD method was applied to three compounds of the abovementioned triazole-based SCO family as well as one mononuclear SCO complex. Due to the exploratory nature of this work, particular attention was focused on the experimental parameters in order to start investigating the relation between the SD protocol and the achieved materials.

## 2. Results and Discussion

### 2.1. Opening Statement on the Tuning of Spray-Drying Parameters

**Figure 1:**
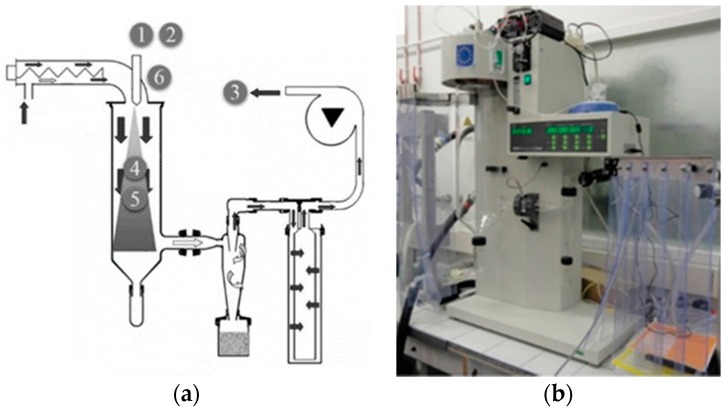
(**a**) Schematic view of the spray-drying (SD) experimental setup. Tunable parameters are positioned and labeled: 1 viscosity of the initial liquid, 2 initial iron (II) and ligand concentrations, 3 suction flow, 4 air flow, 5 drying temperature, and 6 design of the nozzle; (**b**) view of the SD setup Büchi B-290 used in the laboratory.

It is well known that playing with operating parameters of the SD method allows variation of the final product features [33,34,35,36,37]. Figure 1 presents a schematic view and labeling of six experimental parameters. From the literature, the expectations are that increasing parameters 1, 2, or 4 should increase the size of the particles, while increasing parameter 3 decreases it. In addition, playing with 5 and 6 allows one to modulate, in one sense or another, the size and porosity of the NPs. Since it is the first time that SD was used for SCO materials, it has been necessary to play with these parameters to quickly explore the potentiality of the approach. The results shown below present neither an exhaustive nor a systematic study of the SD method applied to the SCO materials design, but a proof of concept of the feasibility to get SCO particles from SD. In the course of this exploration, it has been clear that many parameters, such as 1–6 stated above, may be tuned, but without clear effects on the final products investigated (at least at the level of details targeted here), an exception being parameter 6 as mentioned below. For the same compound, the SCO particle sizes and morphologies obtained are therefore rather similar when parameters 1–5 are modified, and they appear different from the NPs obtained by micelle techniques. Consequently, not all trials corresponding to modification of parameters 1–5 are shown here. 

### 2.2. The SCO Coordination Polymer [Fe(NH_2_trz)_3_]Br_2_·nH_2_O

The coordination polymer [Fe(NH_2_trz)_3_]Br_2_·nH_2_O is known to undergo an abrupt spin-crossover near room temperature with a hysteresis of about 10–15 K, which a priori marked it as a promising SCO material for applicative targets [41,42]. However, many different high-spin (HS) to low-spin (LS) SCO temperatures (T_1/2down_) are reported in the literature, from 284 K to 318 K [14,17,41,42,43]. Although it is not fully understood, it is, however, clear that this temperature appears highly dependent on the synthesis protocol and the number of water molecules within the iron chains; the ratio of water molecules is hardly controlled and even sometimes difficult to estimate [44]. In any case, the relatively low crystallinity of the synthesized powders has prevented accurate characterization of the crystal structure, but the rough structural description confirms the 1D polymeric chains are packed through interactions mediated by the counter-ions and the solvent molecules [22,23].

**Figure 2:**
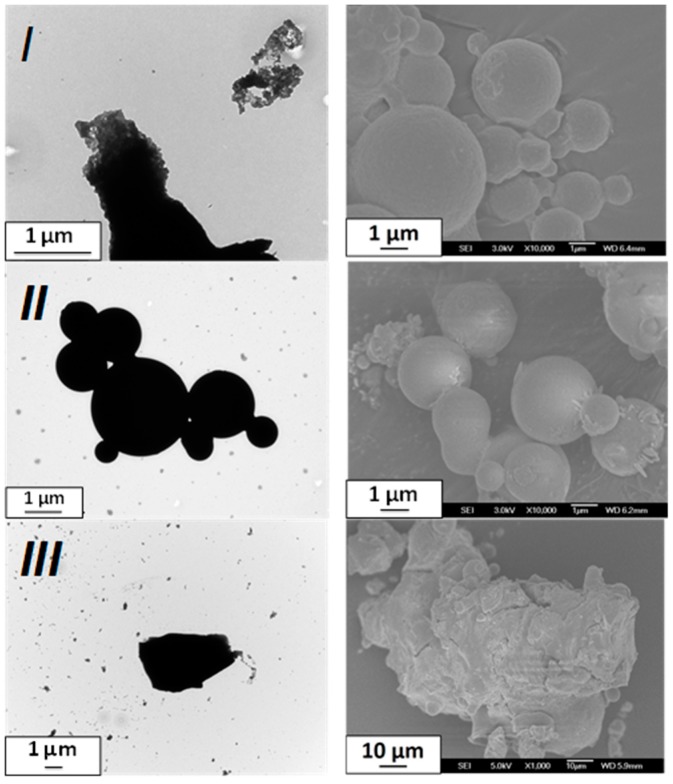
TEM and SEM images of the particles obtained from spray-drying for the selected batches *I*, *II*, and *III* for [Fe(NH_2_trz)_3_]Br_2_·nH_2_O.

**Figure 3:**
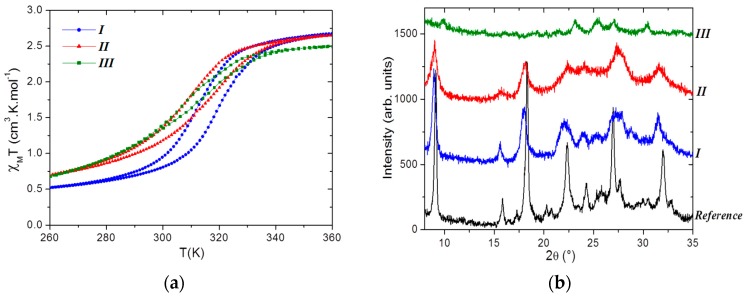
Physical characterizations of batches *I* (blue), *II* (red), and *III* (green) showing (**a**) χ_M_T as a function of temperature and (**b**) the powder X-ray diffraction (PXRD) patterns, including the reference one for [Fe(NH_2_trz)_3_]Br_2_·nH_2_O. Note that while not fully indexed, the latter corresponds to the hexagonal unit-cell with a = b = 19.653(2) Å and c = 7.371(1) Å [23].

Figure 2 shows the resulting particles of three selected trials from a synthesis using the SD method (see Materials and Methods below for details). At first sight, the particles are micro-sized and clearly adopt a spherical shape for *I* and *II*, while *III* shows undefined clusters. All the batches exhibit an SCO in the expected temperature range for this compound, with T_1/2down_ of 316 K, 313 K, and 305 K for *I*, *II*, and *III*, respectively (Figure 3). The SCO is, however, incomplete and more gradual than expected in the three cases with also narrower hysteresis (8 K for *I* and *II*, 4 K for *III*). The color of the samples goes from pink to white, as is usual in the case of the SCO in triazole-based iron coordination polymers.

The powder X-ray diffraction (PXRD) patterns (Figure 3) reveal a very low crystalline quality that gets even worse going from *I* to *III*. The few Bragg peaks that can be identified are compatible with the [Fe(NH_2_trz)_3_]Br_2_·nH_2_O reference, at least for *I* and *II*, while *III* shows some differences. The large width of the Bragg peaks is the mark of nanoscale crystallites. As suspected from microscopy images (Figure 2), this confirms that the micro-sized spherical particles are made by an assembly of nanoscale crystallites. The elemental analysis of *II* (Table 1) only roughly fits for the already known possible chemical compositions. The microanalyses of *I* and *III* clearly show a departure from the expected values, and the synthesis of a new chemical composition or of a mixture cannot be excluded in these cases. The batches *I* and *II* experimentally differ by their experimental protocol (parameters 1–5), while *III* differs from the two others by the use of a three-fluid nozzle instead of a two-fluid one (above parameter 6). It is too early to directly link these differences to the small SCO behavior discrepancies, and it is also obvious that deeper characterizations of the particles are required. Though, this study clearly demonstrates that the SD method can be used to get SCO materials, which was one of the initial target of these first attempts. The particle morphology and the SCO features appear modified in comparison with the reference material.

### 2.3. The SCO Coordination Polymer [Fe(NH_2_trz)_3_](BF_4_)_2_

The coordination polymer [Fe(NH_2_trz)_3_](BF_4_)_2_ undergoes a complete SCO at low temperature with T_1/2down_ = 220 K and a hysteresis of few Kelvin [17]. Reported SCO features for this compound differ a lot, depending on the literature [17,41,42,43,44]. It has been recently demonstrated that this variation was governed by the presence of water in the compound as well as the occurrence of two structural phases [23,44]. Thanks to a careful investigation of the synthesis protocol and the determination of the crystal structure, it is clear that the non-hydrated compound can also be obtained; it will be used as a reference target here. The results for two batches, denoted *IV* and *V*, are reported here.

**Figure 4:**
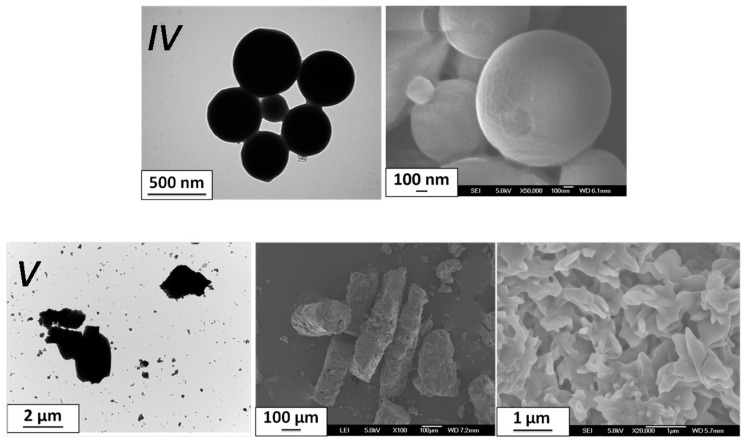
TEM and SEM images of the particles obtained from spray-drying for the selected batches *IV* (**high**) and *V* (**low**) for [Fe(NH_2_trz)_3_](BF_4_)_2_.

**Figure 5:**
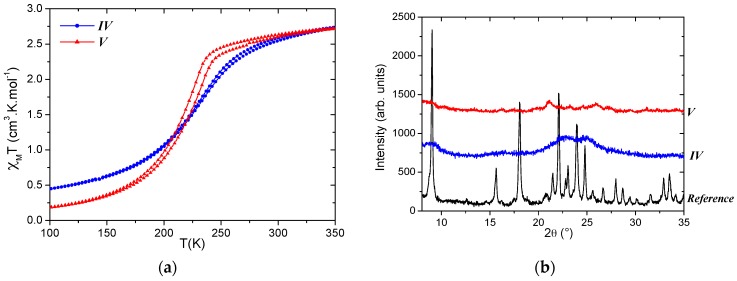
Physical characterizations of batches *IV* (blue) and *V* (red) showing (**a**) χ_M_T as a function of temperature and (**b**) the PXRD patterns, including the reference one for [Fe(NH_2_trz)_3_](BF_4_)_2_. The latter corresponds to the hexagonal unit-cell with a = b = 19.644(1) Å and c = 7.726(1) Å [23].

The SD method leads to micro-sized particles as in the previous case (Figure 4). The particles clearly adopt a spherical morphology for batch *IV* (two-fluid nozzle), while particles aggregate within micro-sized trunk-shaped clusters for batch *V* (three-fluid nozzle). The particle morphologies are therefore reminiscent of the results obtained for the previous compound (batches *I*–*III*) and it seems that the final aspect (sphere or cluster) is somewhat correlated to the choice of the nozzle in the SD protocol (parameter 6). A zoom on the *V* formed clusters reveals undefined-shaped particles of about one micron long (Figure 4). Both batches show an SCO at low temperatures in the expected range (T_1/2down_ = 225 K and 215 K for *IV* and *V* with a hysteresis of 1 K and 6 K, respectively) but with a pronounced gradual character and an incomplete conversion (Figure 5). The color of batch *IV* is as expected for [Fe(NH_2_trz)_3_](BF_4_)_2_, which is white at high temperature and pink-violet below the SCO, contrary to the unusual light-green color of *V*. While [Fe(NH_2_trz)_3_](BF_4_)_2_ powders obtained by classical techniques are rather well crystallized, batches *IV* and *V* show very large Bragg peaks (Figure 5) that do not allow formal confirmation of the nature of the final materials, but indicate nanoscale crystallites. Note that gradual transitions and low crystallinity (i.e., small coherent domains) are known to be linked. The positions of the few observable Bragg peaks for batch *V* are not fully consistent with those expected. In addition, the elemental analysis shows significant differences from the references (Table 2), suggesting that the final product can slightly differ from [Fe(NH_2_trz)_3_](BF_4_)_2_. Though, again, it appears difficult to further characterize the materials, it is clear that SCO compounds have been obtained using the SD method. It is also clear that the morphology of the particles contrasts with the rod-shaped particles usually obtained for the triazole-based SCO compounds. It seems also that though roughly similar, the SCO features show incomplete and more gradual conversion in coherence with the loss of crystallinity. The latter indicates nano-sized coherent domains. The micro-sized particles observed with electronic microscopy are therefore certainly made of an assembly of SCO NPs. 

### 2.4. The SCO Coordination Polymer [Fe(Htrz)_2_(trz)](BF_4_)_2_

The compound [Fe(Htrz)_2_(trz)](BF_4_) (Htrz = 1H-1,2,4-triazole and trz = deprotonated triazolato ligand) is probably the most studied of the SCO coordination polymers for its chemical stability, the very abrupt and large SCO hysteresis loop between 345 K and 385 K, together with clear evidence that it is possible to get rod-shaped particles with a large panel of sizes, from the nano- to the micro-range, in a controlled way [4,5,6,7,8,15,16,17,19,27,28,29,30,31,32,45,46]. Some indications of fatigability have been recently shown after a large number of SCO cycles were applied to the nanorods [7,47]. The [Fe(Htrz)_2_(trz)](BF_4_) crystal structure has been determined in HS and LS and confirmed for a large range of particle sizes, showing first that it crystallizes always in the same orthorhombic phase and then that interchain interactions trigger large structural modifications between the HS and LS crystal packing [20]. Two trials, batches *VI* and *VII*, are reported.

**Figure 6:**
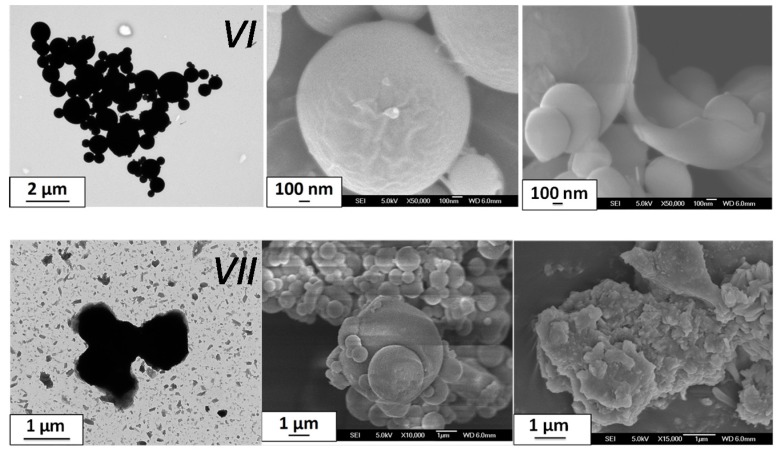
TEM and SEM images of the particles obtained from spray-drying for the selected batches *VI* and *VII* for trials to get [Fe(Htrz)_2_(trz)](BF_4_).

**Figure 7:**
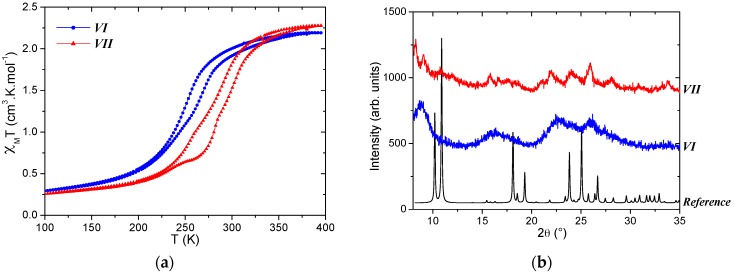
Physical characterizations of batches *VI* (blue) and *VII* (red) showing (**a**) χ_M_T as a function of temperature and (**b**) the PXRD patterns, including the reference one for trials to get [Fe(Htrz)_2_(trz)](BF_4_). The latter corresponds to the orthorhombic unit-cell with a = 17.3100(9) Å; b = 7.3495(4) Å and c = 9.2149(5) Å [20].

The particles obtained by SD for *VI* are clearly micro-sized spheres (Figure 6). Some SEM images of *VI* suggest that the micro-sized particles are indeed hollow spheres. Note that ultrasound treatments of the samples failed to crack the spheres. In the batch *VII* that uses a three-fluid nozzle, the particles aggregate within unclearly shaped clusters. The particles of *VI* and *VII* exhibit a gradual SCO at low temperature, 249 K for *VI* and 283 K for *VII*, with a narrower-than-expected hysteresis, 14 and 10 K, respectively (Figure 7). It is worth recording that the parent compound [Fe(Htrz)_3_](BF_4_)_2_ presents a very abrupt SCO centered at 279 K with a hysteresis width of 6 K [46]. The SCO features obtained here do not fully fit with this behavior, but appear relatively closer to [Fe(Htrz)_3_](BF_4_)_2_ than from the targeted [Fe(Htrz)_2_(trz)](BF_4_) compound. The PXRD patterns confirm the low crystallinity of the particles obtained with SD and here also indicate nano-sized crystallites. The few observable Bragg peaks are not coherent with the well-crystallized reference. In addition, the microanalysis does not fit *VI* and *VII* with one of the known compounds (Table 3). The gradual SCO feature is in line with the poor crystallinity, but the SCO temperatures are lower than expected. Preliminary characterizations seem to indicate that the sample is either a polymorph of the title compound or a new compound. This third study, however, confirms the feasibility of the SD synthesis of SCO material with spherical shapes, but it also confirms the poor crystallinity of the final powders, and thus the difficulties to characterize them. SD is a very violent synthesis that, apparently, does not offer enough time for our materials to organize within large crystallites.

### 2.5. The SCO Mononuclear Complex [Fe(bpp)_2_](NCS)_2_·2H_2_O

The SD method was also tested to get the mononuclear complex [Fe(bpp)_2_](NCS)_2_·2H_2_O (bpp = 2,6-bis(pyrazol-3-yl)pyridine) known for an abrupt SCO without hysteresis at 227 K and its high value of limit temperature, above which the photo-induced HS state is erased, namely T(LIESST), of 73 K [48,49]. Note that these reported values for SCO features are always obtained after an initial heating of the fresh sample at 380 K. The behavior of the nonheated sample is much more intricate, still debated, and is not the topic of this work; therefore, the SCO features shown below are obtained after this initial heating. 

Two batches are reported, denoted *VIII* and *IX*. In this case, however, the SD synthesis protocol is different from the previous ones since the starting material was already a powder of the targeted material [Fe(bpp)_2_](NCS)_2_. The latter is dissolved before the atomization which, in this case, therefore aims to test if the SD treatment allows, or not, to keep the SCO and to challenge potential new SCO features and morphologies.

**Figure 8:**
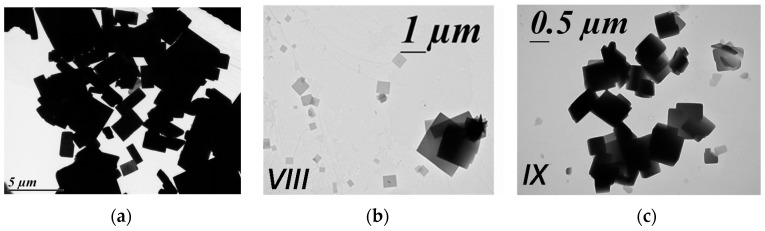
TEM images of the mononuclear SCO [Fe(bpp)_2_](NCS)_2_·2H_2_O particles (**a**) before the SD process and (**b**,**c**) obtained from spray-drying for the for batches *VIII* and *IX*.

**Figure 9:**
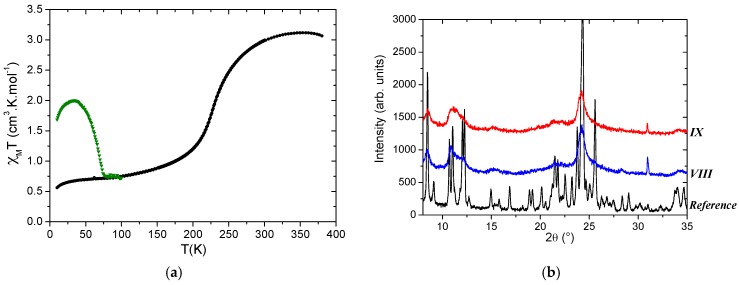
Physical characterizations showing (**a**) χ_M_T as a function of temperature including LIESST effect (green) of batches *VIII* and *IX* and (**b**) the PXRD patterns of batches *VIII* (blue) and *IX* (red), including the reference one for [Fe(bpp)_2_](NCS)_2_·2H_2_O. The latter is known to crystallize in the triclinic unit-cell with a = 8.302(6) Å; b = 8.446(6) Å; c = 21.531(13) Å, α = 78.78(5)°, β = 82.50(5)° and γ = 89.85(4)° [48].

The TEM images shows plate-shaped particles with a large panel of sizes going to less than 100 nm to a few micro-sized ones; this is reminiscent of the morphology obtained by the classical synthesis but with slightly smaller sizes (Figure 8). The batches *VIII* and *IX* adopt exactly the same SCO behavior; that is, a gradual SCO (Figure 9). The magnetic and photomagnetic measurements show an SCO at 227 K and a T(LIESST) at around 70 K for both batches. The thermo- and photo-induced SCO temperatures therefore perfectly match the expected ones for the initial compound. In addition, the elemental analysis of *VIII* and *IX* are clearly in agreement with the chemical composition of the targeted compound [Fe(bpp)_2_](NCS)_2_·2H_2_O (Table 4). PXRD patterns indicate a very bad crystalline quality, the few observable Bragg peaks being in adequacy with the reference. Consequently, here, the SD treatment has allowed obtaining of the anticipated compound in a very quick and easy way. The main difference with the initial material is again, however, the more gradual character of the SCO and the poor crystallinity, both being in adequacy, for the SD-synthesized compound.

## 3. Materials and Methods

### 3.1. Syntheses by Spray-Drying

All the chemicals and solvents were used as purchased. The apparatus used to dry the solutions or suspensions is a Mini Spray Dryer B-290 (BüCHI Labortechnik AG, Flawil, Switzerland) equipped with a two-fluid nozzle (spray cap: 0.7 mm diameter hole) or a three-fluid nozzle (spray cap: fluid (1) 0.7 mm diameter hole/fluid (2) 2 mm diameter hole). Note that the former allows the use of one liquid and one gas, while the latter corresponds to the use of two separate liquids and one gas.

The SD syntheses aimed to obtain the coordination polymers [Fe(NH_2_trz)_3_]Br_2_—batches *I*, *II*, and *III*, [Fe(NH_2_trz)_3_](BF_4_)_2_—batches *IV* and *V*—and [Fe(Htrz)_2_(trz)](BF_4_)—batches *VI* and *VII*—as well as the mononuclear complex [Fe(bpp)_2_](NCS)_2_—batches *VIII* and *IX*. For a same target, the batches refer to different experimental conditions. In all below cases, an atomizing airflow rate between 8 and 10 L·min^−1^ was used.

SD synthesis of *I*: an aqueous solution of NH_2_trz (0.15 M; 10 mL) was added at a flow rate of 5 mL/min to an aqueous solution of FeBr_2_ (0.05 M; 10 mL) containing 0.01 g ascorbic acid. The medium was stirred at R.T. during 15 min, then stored at 5 °C during 1 h. The resulting transparent solution was then spray-dried using the two-fluid nozzle (combined with a peristaltic pump) at a feed rate of 6 mL·min^−1^ (20%), an inlet air temperature of 90°C, and an aspirator rate of 60% (25 m^3^·h^−1^); 20 mg of a pink powder was collected.

SD synthesis of *II*: an aqueous solution of NH_2_trz (0.15 M; 20 mL) and an aqueous solution of FeBr_2_ (0.05 M; 20 mL) containing 0.02 g ascorbic acid were separately pumped, then joined in a single tube to feed the spray-dryer via the two-fluid nozzle. The feed rate was 6 mL·min^−1^ (20%), the inlet air temperature was 90 °C, and the aspirator rate was 70% (28 m^3^·h^−1^); 300 mg of a white powder was collected, which became dark-pink after one night.

SD synthesis of *III*: an ethanolic solution of NH_2_trz (1.2 M; 10 mL) and an aqueous solution of FeBr_2_ (0.4 M; 10 mL) containing 0.03 g ascorbic acid were separately pumped to feed the spray-dryer via a three-fluid nozzle (in this case, two separate peristaltic pump were used, having the same feed rate). The parameters used are: inlet air temperature of 100 °C and an aspirator rate 100% (38 m^3^·h^−1^). At the end, 180 mg of a white powder was collected, and the powder became dark-pink after one night.

SD synthesis of *IV*: an aqueous solution of NH_2_trz (0.06 M; 20 mL) was added at a flow rate of 5 mL/min to an aqueous solution of Fe(BF_4_)_2_·6H_2_O (0.05 M; 20 mL) containing 0.02 g ascorbic acid. The medium was then stirred at R.T. during 15 min, and then stored at 5 °C during 1 h. The resulting transparent solution was then spray-dried using a two-fluid nozzle (combined with a peristaltic pump) at a feed rate of 5 mL·min^−1^ (20%) and an aspirator rate of 80% (33 m^3^·h^−1^); 100 mg of a white powder was collected, and the powder became very-light-pink after one night.

SD synthesis of *V*: an ethanolic solution of NH_2_trz (1.2 M; 10 mL) and an aqueous solution of Fe(BF_4_)_2_·6H_2_O (0.4 M; 10 mL) containing 0.03 g ascorbic acid were separately pumped to feed the spray-dryer via a three-fluid nozzle. The parameters used were: the inlet air temperature fixed at 100 °C and an aspirator rate of 100% (38 m^3^·h^−1^); 480 mg of a light-green/white powder was collected.

SD synthesis of *VI*: an ethanolic solution of Htrz (0.06 M; 20 mL) was added at a flow rate of 5 mL/min to an aqueous solution of Fe(BF_4_)_2_·6H_2_O (0.02 M; 20 mL) containing 0.01 g ascorbic acid. The medium was stirred at R.T. during 15 min, then stored at 5 °C during 1 h. The resulting transparent solution was spray-dried using a two-fluid nozzle (combined with a peristaltic pump), an inlet air temperature of 80 °C, and an aspirator rate of 80% (33 m^3^·h^−1^); 80 mg of a white powder was collected, which became pink after one night.

SD synthesis of *VII*: an ethanolic solution of Htrz (0.06 M; 10 mL) and an aqueous solution of Fe(BF_4_)_2_·6H_2_O (0.02 M; 10 mL) containing 0.01 g ascorbic acid were separately pumped to feed the spray-dryer via a three-fluid nozzle. The parameters used were: the inlet air temperature fixed at 100 °C and an aspirator rate of 100% (38 m^3^·h^−1^); 20 mg of a white powder was collected, which became pink after one night.

SD leading to *VIII*: 150 mg of [Fe(bpp)_2_](NCS)_2_ was dissolved in a mixture of 30 mL ethanol and 10 mL water. The resulting solution was then spray-dried using a two-fluid nozzle, a feed rate of 5 mL·min^−1^, an inlet air temperature of 90 °C, and an aspirator rate of 70% (28 m^3^·h^−1^). At the end of aspiration, 25 mg of a dark-red powder was collected.

SD leading to *IX*: the initial solution was based on 150 mg of [Fe(bpp)_2_](NCS)_2_ in a mixture of 60 mL ethanol and 20 mL water, leading to a concentration lower than that for *VIII*. The same experimental parameters as *VIII* were then used for the atomization itself. Finally, 25 mg of a dark-red powder was collected.

### 3.2. Physical Characterizations

Powder X-ray diffraction measurements have been performed using a PANalytical X′Pert PRO diffractometer (Cu-Kα, X′Celerator detector) (Almelo, The Netherlands) within the range 8°–80° (2θ) using 60 s exposure with 0.017° steps. The Cu-Kα radiation was generated at 45 kV and 40 mA (λ = 0.15418 nm). The samples were put on sample holders made of aluminum alloy and flattened with a piece of glass.

Scanning electron microscopy (SEM) images were taken with a JEOL 6700F (Akishima, Tokyo, Japan) with 5 kV tension; the latter being low in order not to destroy the investigated materials. Transmission electron microscopy (TEM) images were acquired using a HITACHI H7650 (Hitachinaka, Japan), with a high-resolution mode and a tension of 60 kV.

Elemental analysis (CHN) of *I* to *IX* were investigated using a Thermo Fisher flash EA-1112 (Waltham, MA, USA).

Variable temperature magnetic data for the samples were collected using a Quantum Design MPMS-7S magnetometer (San Diego, CA, USA) under a field of 5000 Oe. Measurements were performed every Kelvin in settle mode for all batches except batch *III* (every 2 K). The thermal cycles were achieved with a rate of 1 K·min^−1^. T (LIESST) measurements were achieved following a well-known procedure [50].

## 4. Conclusions

This exploratory study of the spray-drying (SD) method to get SCO materials brings much learning. Above all, it shows the feasibility to get SCO compounds through the SD method. Then, if all of the four synthetized materials undergo an SCO, the features of the latter appear somewhat different to those of the referenced samples obtained by classical synthesis (bulk materials) or micelle techniques (NPs). For the coordination polymers, the materials achieved by SD exhibit an SCO with narrower hysteresis widths and with a much more pronounced gradual character, sometimes leading to incomplete SCO. These features are in line with a strong decrease of the crystallinity indicating nanoscales coherent domains. It seems also that, at least in one case, a new phase or a novel compound has been obtained using SD; which incidentally opens possible routes to the design of original SCO materials. The initial goal, which was to get new morphologies, is achieved since, clearly, upon certain experimental conditions (as for example the use of a two-fluid nozzle), spherical particles have been designed, contrasting with the rods achieved from other methods (notably for [Fe(Htrz)_2_(trz)](BF_4_)). For the mononuclear complex, the compound treated by the SD method led to the same SCO features as the reference compound, despite the noticeable difference of a more gradual SCO. In all above cases, SCO materials with gradual SCO and low crystallinity are obtained; however, the synthesis was able to take advantage of the quickness and easiness of the SD process.

Establishing correlations between the SD protocols used in investigated batches and the various particles features would be premature. These results must be seen as an initial contribution, a proof of concept, to the design of SCO particles using spray-drying. One very interesting perspective to this work is certainly the use of SD to encapsulate the SCO nanoparticles with, for example, an organic polymer.

## Figures and Tables

**Table 1:** Elemental analysis (CHN) for batches *I*, *II*, and *III*, including the expected values for [Fe(NH_2_trz)_3_]Br_2_·nH_2_O with *n* = 0, 1, 2, and 3.

Sample	% N	% C	% H
batch *I*	30.32	15.48	3.24
batch *II*	32.38	15.38	3.12
batch *III*	37.36	16.17	3.39
[Fe(NH_2_trz)_3_]Br_2_	35.92	15.40	2.59
[Fe(NH_2_trz)_3_]Br_2_·H_2_O	34.59	14.83	2.90
[Fe(NH_2_trz)_3_]Br_2_·2H_2_O	33.35	14.30	3.20
[Fe(NH_2_trz)_3_]Br_2_·3H_2_O	32.20	13.81	3.48

**Table 2:** Elemental analysis (CHN) for batches *IV* and *V* with expected values for [Fe(NH_2_trz)_3_](BF_4_)_2_.

Sample	% N	% C	% H
batch *IV*	28.25	17.77	2.84
batch *V*	36.99	16.00	2.91
[Fe(NH_2_trz)_3_](BF_4_)_2_	34.89	14.96	2.51
[Fe(NH_2_trz)_3_](BF_4_)_2_·2H_2_O	32.47	13.92	3.11

**Table 3:** Elemental analysis (CHN) for batches *VI* and *VII* with expected values for trials to get [Fe(Htrz)_2_(trz)](BF_4_).

Sample	% N	% C	% H
batch *VI*	23.84	16.13	3.15
batch *VII*	24.45	15.70	2.97
[Fe(Htrz)_2_(trz)](BF_4_)	36.10	20.66	2.29
[Fe(Htrz)_2_(trz)](BF_4_)·3H_2_O	31.29	17.89	3.38
[Fe(Htrz)_3_](BF_4_)_2_	28.87	16.51	2.06

**Table 4:** Elemental analysis (CHN) for batches *VIII* and *IX* with the expected values for [Fe(bpp)_2_](NCS)_2_·2H_2_O.

Sample	% N	% C	% H
batch *VIII*	25.77	45.73	3.24
batch *IX*	25.98	46.12	3.26
[Fe(bpp)_2_](NCS)_2_·2H_2_O	26.66	45.72	3.52
